# Born Too Soon: Progress and priorities for respectful and rights-based preterm birth care

**DOI:** 10.1186/s12978-025-02042-w

**Published:** 2025-06-23

**Authors:** Mary Kinney, Elena Ateva, Olive Cocoman, Marta Schaaf, Phillip Wanduru, Merette Khalil, Emma R. Sacks, Regina Tames, Denise Suguitani, Marcus Stahlhofer, Jaideep Malhotra, Petra Ten Hoope Bender

**Affiliations:** 1 School of Public Health, University of the Western Cape, Bellville, South Africa; 2 Global Surgery Division, Department of Surgery, University of Cape Town Faculty of Health Sciences, Observatory, Western Cape South Africa; 3 White Ribbon Alliance, Washington, D.C USA; 4 Department of Maternal, Newborn, Child and Adolescent Health and Ageing, World Health Organization, Geneva, Switzerland; 5 Department of Infections Disease Epidemiology and International Health, London School of Hygiene and Tropical Medicine, London, UK; 6 Institute of Global Health, United Nations University, Kuala Lumpur, Malaysia; 7 Amnesty International, London, UK; 8 Department of Health Policy, Planning and Management, School of Public Health, Makerere University, New Mulago Hill Road, Kampala, Uganda; 9 Department of Global Public Health, Karolinska Institutet, Stockholm, Sweden; 10 YourEgyptianDoula, Cairo, Egypt; 11 Department of International Health, Johns Hopkins School of Public Health, Baltimore, MD USA; 12 Global Partnerships, Global Fund for Women, Mexico City, Mexico; 13 Brazilian Parents of Preemies’ Association, Porto Alegre, Brazil; 14 ART Rainbow IVF, Agra, India; 15 United Nations Population Fund, Office of Geneva, Geneva, Switzerland

**Keywords:** Human rights, Preterm birth, Respectful care, Family-centred care

## Abstract

**Progress:**

Human rights related to preterm birth encompass access to respectful, evidence-based care; informed consent; protection from discrimination, detention, and unnecessary separation of mother and newborn; and broader social entitlements, such as parental leave and early disability support. Since the 2012 *Born Too Soon* report, global recognition of these rights has expanded through international treaties, global guidelines, national legal reforms, and social movements. Demand for respectful care, including respectful maternity care and family centred care, has led to its incorporation into global guidelines and policies and a greater evidence-base. However, persistent challenges, such as workforce shortages, discriminatory policies, and the erosion of sexual and reproductive rights, continue to threaten progress.

**Programmatic Priorities:**

Ensuring respectful and rights-based preterm birth care requires coordinated action across the continuum of care and across sectors, with the mother–baby dyad at the centre. Programmatic priorities at the individual level include implementing respectful maternity care and family-centred care. Ensuring high-quality, respectful care demands that providers themselves are supported, protected, and empowered to deliver such care. Their well-being is a critical enabler of the rights of patients and an essential component of effective, compassionate service delivery. At the facility-level, health systems must be purposefully designed to safeguard the fundamental human rights of the individuals with them, both care seekers and care providers. Implementing respectful, rights-based care relating to preterm birth requires structural and social changes, as well as robust data systems for accountability. Multi-stakeholder action requires strengthening accountability mechanisms at all levels and partnering with those affected by preterm birth—particularly women, families and healthcare providers—in policy processes, and the design, implementation and monitoring of care. At national-level, action requires the adoption, implementation and monitoring of international and regional human rights instruments, with multisectoral collaboration and social mobilization where violations continue.

**Pivots:**

To operationalize respectful and rights-based care for preterm birth, four primary shifts are needed: scale up respectful care; empower and partner with women and families; address the shortage of healthcare providers and protect their rights; and strengthen policy action and accountability.

**Supplementary Information:**

The online version contains supplementary material available at 10.1186/s12978-025-02042-w.

## Plain language summary

Since the release of the 2012 Born Too Soon report, there have been significant improvements in global policies and actions regarding preterm birth, with a stronger focus on human rights. The right to respectful care for women and families is an increasing priority, along with the rights of healthcare workers and the need for greater accountability.

Priority actions are necessary across multiple tiers, all grounded in human rights standards and principles. At the national level, governments are responsible for adopting, implementing and monitoring human rights laws related to preterm birth. Public participation is key in holding policymakers accountable. In hospitals and clinics, partnering with women and parents of preterm babies has been shown to improve the quality of care. Practices that disrespect women and their babies—such as lack of privacy during childbirth, unnecessary C-sections, or separating mothers from their babies—must be eliminated to ensure more respectful, family-centered care.

Creating a supportive work environment and enhancing the competencies of healthcare providers are also critical components. Women, their partners, and healthcare workers need to be empowered with the knowledge and tools to make informed decisions and advocate for respectful treatment.

Ensuring respect for human rights in preterm birth care requires action at multiple levels, from families and healthcare professionals to government policies. Strong partnerships between all stakeholders—mothers, healthcare workers, communities, and legal systems—are crucial. National laws and healthcare systems must be structured to integrate human rights principles at every level. Furthermore, partnering with advocates, civil society, and community-based initiatives ensures a holistic approach that addresses preterm birth in a rights-centred manner. Protecting healthcare workers' rights at work and setting up clear accountability systems are crucial for achieving lasting improvements in preterm birth care.

**Supplementary Information:**

The online version contains supplementary material available at 10.1186/s12978-025-02042-w.

## Key findings

### Progress

Human rights law and global health guidance documents have increasingly upheld the rights of women, babies, parents and families, and healthcare providers, including advances in the respectful care agenda.

### Priorities

Ensuring rights and respect in preterm birth care requires action across the continuum of care, across sectors, and with strong partnerships between the mother–baby dyad and healthcare providers, as well as the families, communities and systems that support them.

### Pivots

To operationalize respectful and rights-based care for preterm birth, we need to: scale up respectful care; empower and partner with women and families; address the shortage of healthcare providers and protect their rights; and strengthen policy action and accountability.

## Main Body

### Introduction

This paper is part of the Born Too Soon supplement, developed from the report “Born Too Soon: Decade of action on preterm birth,” published in 2023 as part of a movement to prevent and improve care related to preterm birth [1]. The paper focuses on how to embed respectful and rights-based preterm birth care at the core of policy, service, and interaction. It first defines human rights related to preterm birth, and thereafter reflects on the progress (policy shifts and rights recognition), recommended programmatic priorities (key issues across systems), and pivots (implementation-focused solutions to be prioritized). The paper is a narrative commentary, synthesizing evidence from the Born Too Soon report and from new data, literature reviews, and case studies that emphasize policy, implementation, and community perspectives. Additional background to the Supplement can be found in the first paper in this series [2].

### Preterm birth and human rights

**Fig. 1:**
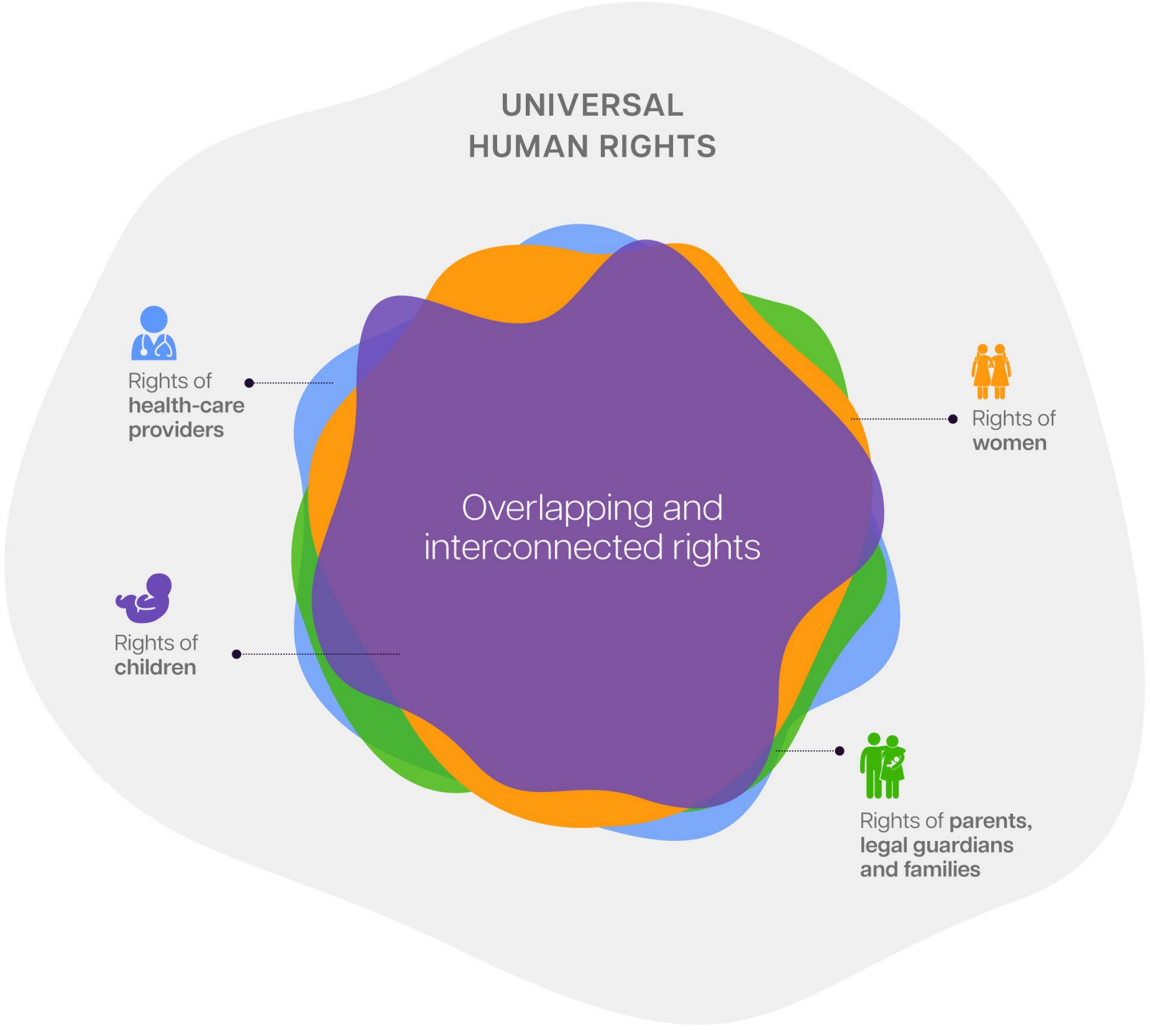
The overlapping human rights of women, newborns, families and healthcare providers

Article 1 of the Universal Declaration of Human Rights states that “all human beings are born free and equal in dignity and rights.” [3] Multiple international conventions incorporate human rights pertinent for preterm babies, their families and their healthcare providers. These include access to respectful, evidence-based care; right to informed consent; protection from discrimination, separation of mother and newborn, or detention; and broader social entitlements such as parental leave, social protection, and early intervention for disability. Healthcare providers have specific rights—to decent working conditions, fair pay, and unionization—yet many lack the resources, support, and recognition needed to provide respectful care [4–7]. Figure 1 illustrates how human rights between those affected by preterm birth mostly intersect and overlap; yet, certain rights are specific to particular populations, or contingent on specific circumstances. Appendix 1 provides a mapping of key human rights conventions relevant preterm birth with illustrative examples of these rights.

### Progress

#### Progress on rights: Laws, policies and social movements

**Fig. 2:**
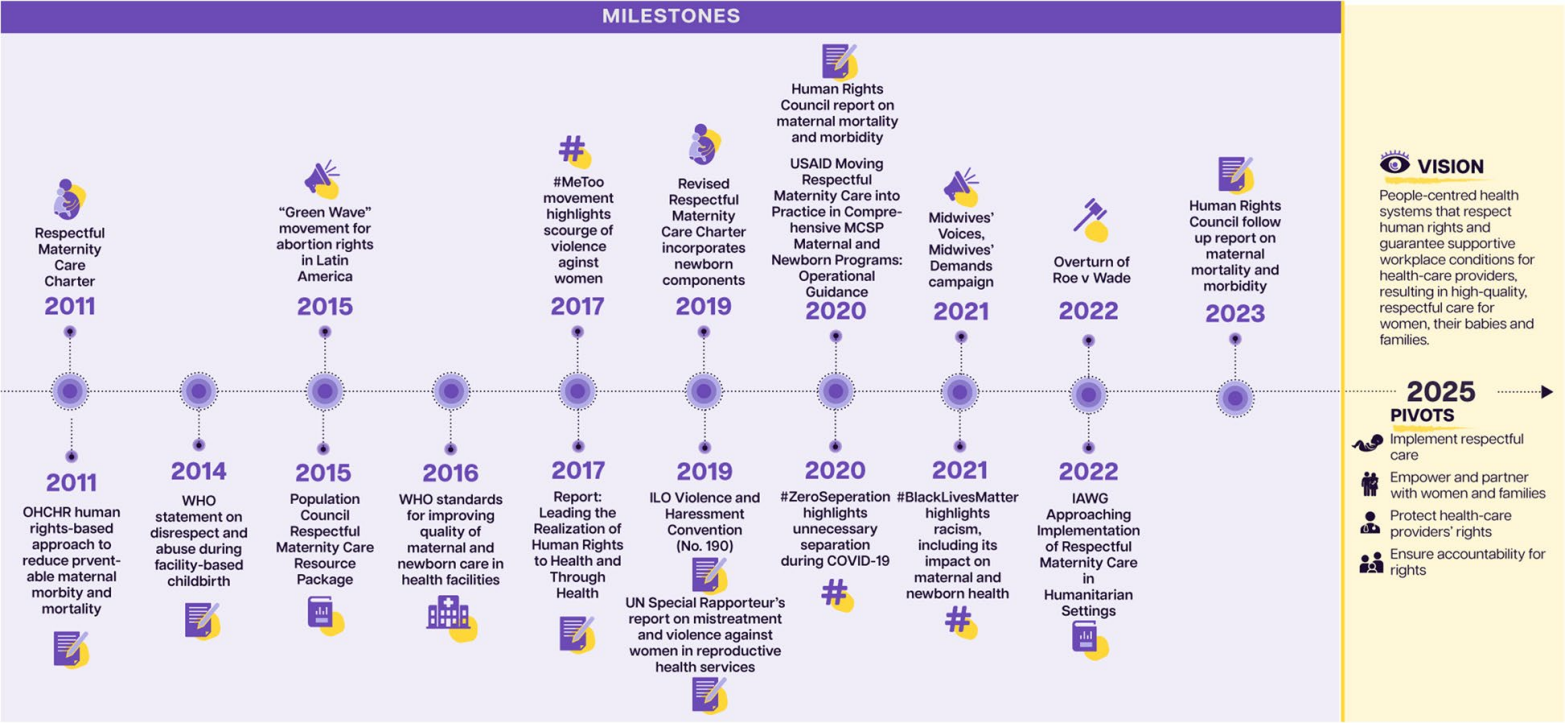
Timeline of key milestones for rights and respect related to preterm birth

Human rights law and global health guidance documents have increasingly recognized that the rights relating to preterm birth are critical for good health and wellbeing, stronger health systems and societal progress in the past century [8]. Since the publication of the 2012 *Born Too Soon* report [9], the global human rights landscape as it pertains to preterm birth has seen major advancements in policies, guidelines and action **(**Fig. 2) [10–16]. The Sustainable Development Goals (SDGs) in 2015 and the supporting *Global Strategy for Women’s, Children’s and Adolescents’ Health* (2016–2030) focussed more strongly on rights than previous global goals and strategies [17], including the establishment of a high-level working group on health and human rights of women, children and adolescents [18]. However, these global frameworks neglected specific mention of preterm birth, thereby inhibiting visibility, which shapes political attention and investment to advance action.

United Nations (UN) human rights treaty-monitoring bodies, such as the Convention on the Elimination of All forms of Discrimination Against Women, the Convention on the Rights of the Child and the International Covenant on Economic, Social and Cultural Rights, have continued to elaborate on the rights of women and newborns, and to clarify state obligations for realizing these rights, such as measures to ensure universal access to birth registration. [19, 20]. In addition, the UN Human Rights Council has acknowledged technical guidance documents on the application of a human rights-based approach to the elimination of preventable maternal mortality and morbidity, and child mortality [21, 22]. Recent human rights instruments, such as the Convention on the Rights of Persons with Disabilities (2006), articulate rights relating to preterm birth [8]. More recent UN General Assembly resolutions on human rights promote factors relevant to preterm birth, such as recognizing the right to a healthy environment (2022) [23], the realization of which would be a significant contribution to the prevention of prematurity resulting from air pollution and other environmental concerns [24, 25].

The rights of healthcare providers have gained growing attention over the past decade, particularly in humanitarian settings and during COVID 19 where many worked under unsafe, overstretched conditions [26, 27]. The International Labour Organization’s Convention No. 190, in 2019, marked a milestone, as the first international instrument to affirm the right to work free from violence and harassment, including gender-based violence [13]. Severe health workforce shortages and capacity—especially in training, support, retention, and access to essential supplies—undermine the care of preterm babies [1, 28]. Recent campaigns, such as *Midwives’ Voices, Midwives’ Demands* (2022), have highlighted the needs and voices of frontline workers [15].

**Fig. 3:**
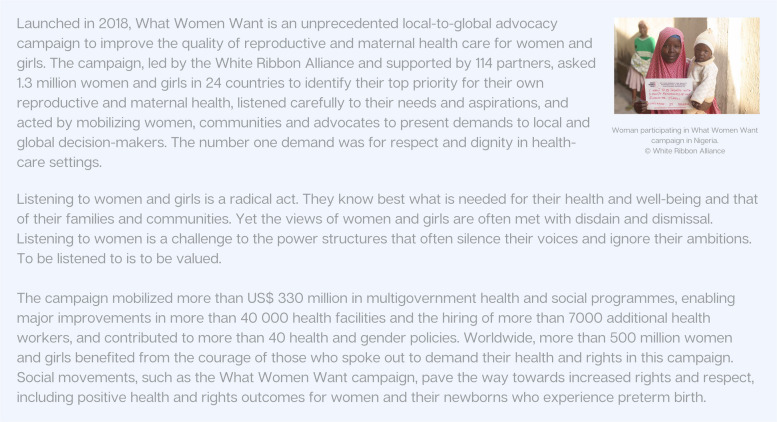
What Women Want: the power of participation. Source: White Ribbon Alliance [14]

Progress in national policies vary. Legal rulings in countries, like Mexico and at the European Court of Human Rights, have upheld patient rights to informed consent and privacy in childbirth [29]. At the same time, significant setbacks—such as the overturning of Roe v. Wade in the United States—have threatened reproductive rights [30]. Some countries have advanced by tracking mistreatment in reproductive health services and taking steps to address it [20]. Global civil society campaigns like “What Women Want” have amplified the demands of women and families, prompting professional bodies to commit to more respectful, dignified care (Fig. 3) [14, 31]. National and regional social movements such as Black Lives Matter, #MeToo, and “The Green Wave” in Latin America have fostered new dialogue and legal reforms related to justice and reproductive rights [2, 32]. The rise of health policy and systems research approaches in maternal and newborn health has further enabled analysis of power dynamics and accountability mechanisms [33].

Sexual and reproductive health and rights (SRHR) are increasingly under threat, with restrictive policies limiting access to services, reducing obstetric providers, and narrowing choices for pregnancy and birth [34, 35]. These setbacks heighten risks of gender-based violence, unsafe abortion, and poor obstetric care, contributing to preventable maternal and newborn deaths [36]. Conflicts and humanitarian crises further endanger women and preterm babies, with growing attacks on health workers and facilities [25, 37]. Gender power imbalances persist across health systems: midwives and nurses—mostly women—are undervalued and under-resourced, and birthing individuals often face disempowering, hierarchical care. Likewise, unequal caregiving burdens, over-medicalization, and pregnancy-related violence undermine respectful, rights-based care [38–40]. Limited progress on the systemic gendered barriers hinders progress for preterm birth outcomes [41].

### Progress on respectful care

**Fig. 4:**
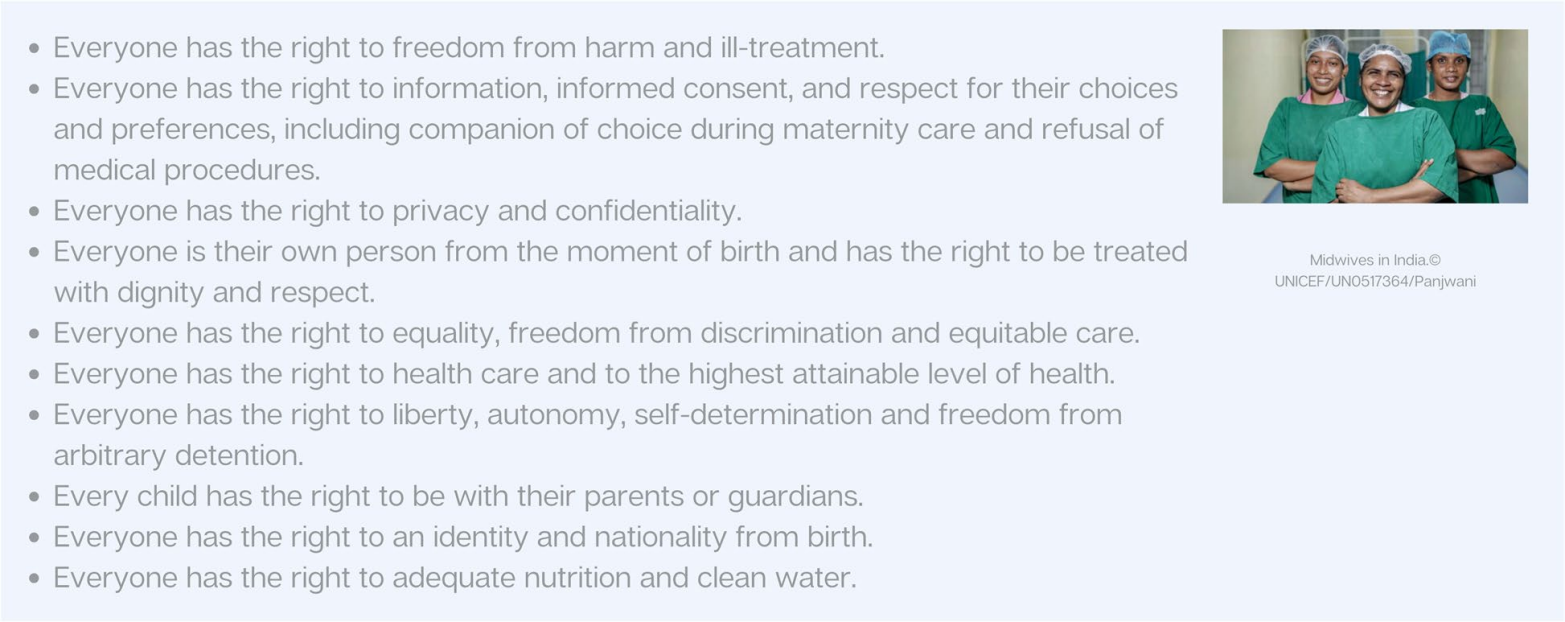
Respectful Maternity Care Charter: the universal rights of women and newborns. Source: White Ribbon Alliance. [11]

In 2016, the World Health Organization (WHO) explicitly incorporated "experience of care"as a core component of quality alongside the “provision of care” in maternal and newborn health guidelines [42]. This marked a paradigm shift acknowledging that how care is delivered (i.e., interpersonal, emotional, respectful aspects) is as important as what care is delivered (i.e., technical quality) [12]. In the past decade, WHO maternal and newborn health related guidelines placed respectful care on equal footing with evidence-based clinical care [2]. Instrumental in catalysing this shift was the advancement of Respectful Maternity Care Charter (RMC), first established in 2011 and then updated in 2019 to include some fundamental rights of newborns (Fig. 4) [11].

WHO defines Respectful Maternity Care as “care organized for and provided to all women in a manner that maintains their dignity, privacy and confidentiality, ensures freedom from harm and mistreatment, and enables informed choice and continuous support during labour and childbirth.” [43] The content of RMC is highly contextual and should be defined by the recipients of healthcare services: women themselves. The growing literature on the mistreatment of women and healthcare providers in maternity settings reinforces the urgency of integrating RMC into health system standards [38, 44–47]. Resource materials and operational guidance to support implementation of RMC [48, 49], set out effective approaches to enhance and uphold RMC [50, 51], including in humanitarian settings [52].

Family-centred care (FCC) is another term relating to “respectful care” often used in preterm birth care, which has also seen a growing body of evidence on improved health outcomes in the past decade, further details under programmatic priorities [16, 53–56]. The demand for FCC was evident in 2020 through the “zero separation” campaign, which responded to restrictive visitation policies during the COVID-19 pandemic that separated parents from their preterm babies [16].

### Programmatic priorities

**Fig. 5:**
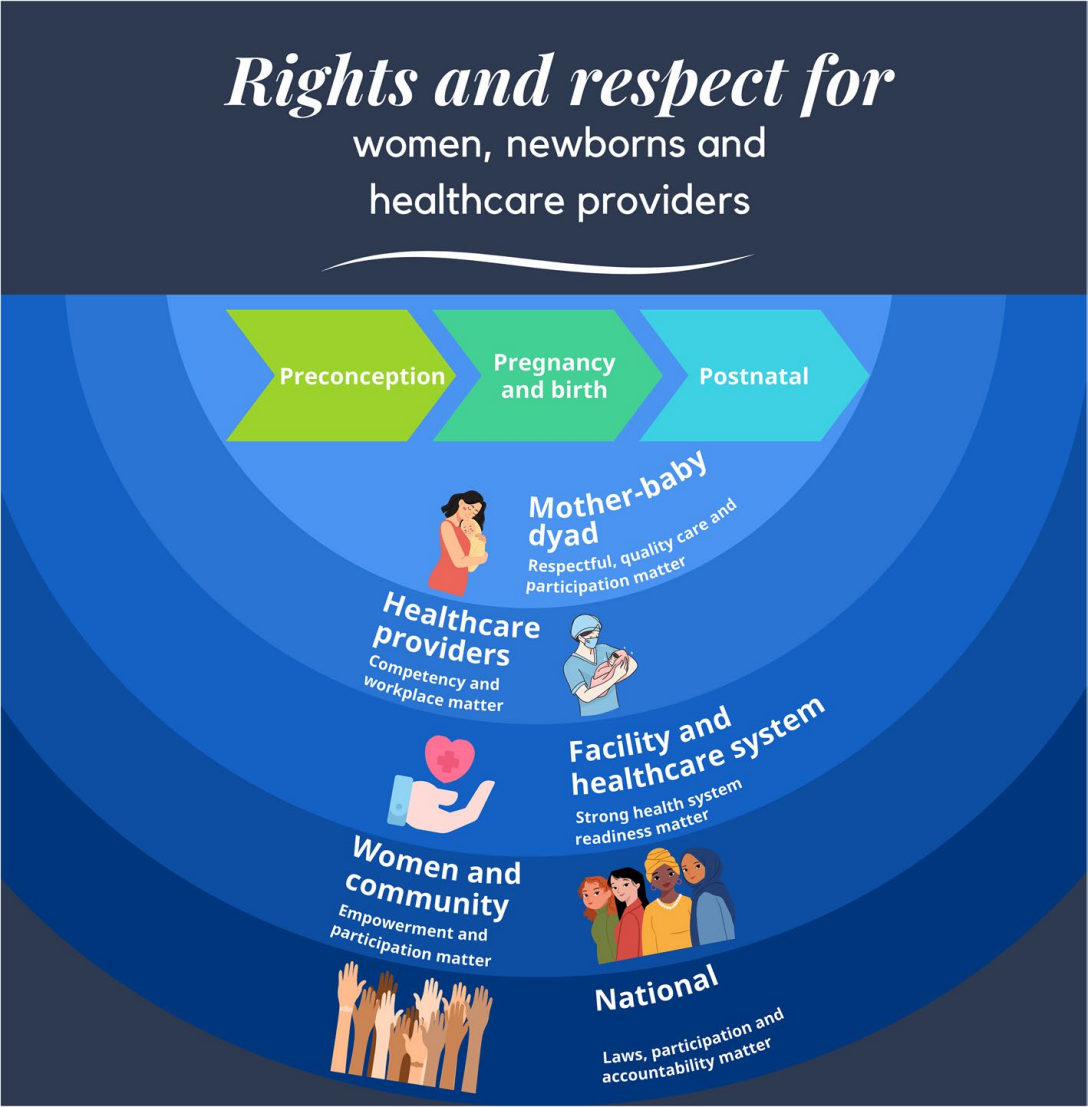
Ecological model as applied to the continuum of rights and respect relate to preterm birth

Ensuring rights and respect in preterm birth prevention and care requires action across the continuum of care, across sectors, and with strong partnerships between the mother–baby dyad and healthcare professionals, and the families, communities and systems that support them. Programmatic priorities building from evidence and country experiences are presented by levels of an ecological framework shown in Fig. 5. [45, 57–60] While priority actions at different levels vary, all must be rooted in human rights standards and principles, including equality and non-discrimination, participation, transparency, empowerment, international cooperation and assistance, accountability, and the best interests of the child [61]. The framework positions the mother-baby dyad at the centre of the care reflecting a person-centred approach for preterm birth action.

### The mother–baby dyad: respectful, high-quality care and participation matter

Firstly, respecting and supporting the mother–baby dyad helps to fulfil the rights of both, and benefiting both, physiologically and emotionally. Caring for the mother and preterm baby as a dyad involves enabling evidence-based practices, such as skin-to-skin contact, Kangaroo care and breastfeeding. [62–64]. At the individual level for mothers and babies, autonomy and influence over decisions regarding the pregnancy and birth are important. While fathers, partners or other caregivers can play a critical role in both supporting the new mother and caring for the baby, mother’s voices need to be heard at all levels of the health system. The contributions of fathers and partners to caregiving also have the benefit of helping them to bond with their preterm babies and to embrace their caregiver roles [65].

Secondly, every accommodation should be made to minimize unwanted separation and/or ensure that separation is never necessary. Any instance of separation needs to be done with the consent of the mother. Additionally, the health care system must enable care for sick mothers together with their preterm baby, ensuring zero separation. When the typical attachment process is interrupted, preterm babies and parents are at risk for short- and long-term health and social problems. For the parents, separation can cause negative health effects, including feelings of shame and guilt, acute or chronic stress disorder, increased maternal and paternal depression, less synchronous behaviours between parents and infants, decreased infant alertness, and decreased parent self-efficacy and confidence in taking care of their child [66–68].

Thirdly, FCC is a core programmatic priorities that requires collaboration between healthcare providers, patients and their families [69]. FCC is rooted in human rights, such as dignity, information sharing, and participation [70]. In preterm care, FCC approaches consider the parents and family to be central to a child’s well-being, a paradigm shift in many neonatal units whereby the child must no longer be seen as just a single individual patient [71, 72]. Adopting FCC approaches in preterm care has proven benefits for newborns and parents in both low- and high-income settings, [16, 53–56], with a multitude of evidence-based interventions [73], and is recommended in the new WHO guidelines on the care of preterm and low-birth-weight babies [74]. For example, parental involvement during inpatient neonatal intensive care improves newborn outcomes, including weight gain and increased frequency of exclusive breastmilk feeding [53, 75].

Mental health support for affected parents is another priority. Parental depression and anxiety may contribute to abuse and neglect of the child which can have a myriad of long-lasting detrimental risks to the child’s development, and the child’s social and cognitive growth [76]. Communication and counselling, effective provider–parental engagement and supportive work environments can reduce parental stress and anxiety and uphold parental rights [59]. Mental health support may also include bereavement care in cases of stillbirth or neonatal death, and counselling on handling medical complexity or post-traumatic stresses when the preterm baby survives.

**Fig. 6:**
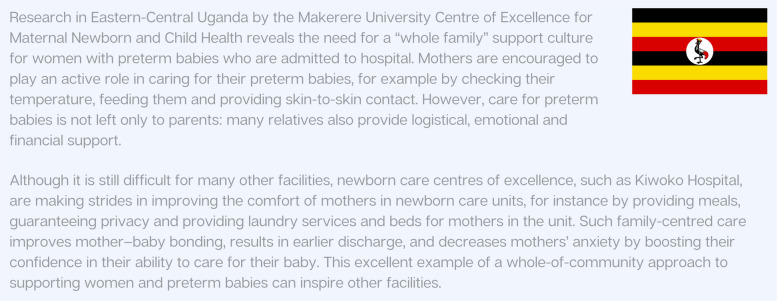
Whole-of-community approach to caring for preterm babies in Uganda

Women especially, but also other affected parents and families, need knowledge, confidence and skills to advocate for respectful care, as well as security against retaliation for speaking out. Empowering women, particularly, is a priority to ensure that they can make effective decisions relating to their health and that of their preterm baby, and seek mental health services when needed. Research and monitoring tools already exist to measure the level of respect for women in maternity care settings, and new tools are under development which incorporate questions about respectful care of the mother–baby dyad in the postnatal period [77]. Interventions to improve the experience of families with babies in intensive care have been identified [67]; however, further research is needed on implementation, especially in low-resource settings. Implementation of FCC models may vary by context [78], and Fig. 6 presents one such example from Uganda.

### Healthcare providers’ competency and workplaces matter

Healthcare providers at the frontline provide clinical care [71] and must also prepare and support parents and families in caring for their preterm babies in hospital and after discharge [79]. To do this respectfully, they need an enabling environment, including adequate salaries, professional development, good supervision, and safety and security [80]. Competencies in evidence-based care are critical —including the latest clinical guidelines, decision-making skills, and critical thinking—so providers can confidently deliver high-quality, respectful, and effective interventions. This requires both preservice education and continuous professional development that reinforces both clinical and interpersonal skills and self-awareness of their own rights. Special training relating to preterm birth may focus, for example, on: birthing companions; communication with affected parents and families to understand their preferences; and presentation of care options in informed and non-judgemental ways. Providers, including midwives, may also need emotional and psychological support to cope with adverse outcomes, such as preterm birth and stillbirth.

Understanding the needs of healthcare providers, both for themselves and to provide high-quality, respectful care to patients, is vital for improving the care of preterm babies and their families. Involving health care staff in quality improvement activities, interpreting and acting on facility data, can help create a culture of and collaboration. Engaging healthcare providers in quality improvement activities, enhances the quality of care and strengthens their commitment to continuous improvement and patient-centred care [81, 82].

### Facility level: strong health-system readiness matters

The health system, including all levels of health facilities, plays a critical role in promoting respectful care and creating a supportive environment for women, preterm babies, parents and healthcare providers. Over the past decade, multiple global guidance documents have been developed to support health facilities in operationalizing a rights and respect-based approach [4, 48, 57]. Long-standing priorities for respectful care at facilities include ensuring privacy during childbirth and quality care for preterm babies and their parents.

Health facility policies must include the elimination of routine practices linked to disrespect, such as unnecessarily breaking the mother–baby dyad or non-medically advised caesarean sections, which can also exacerbate the prevalence of preterm birth [83]. Facility policies need guided by the Convention on the Rights of the Child, ensuring the best interests of the child are considered in treatment plans [8].

There should be no facility policy or practice of detention for unpaid bills, as this is common especially for preterm babies that may require specialized care [84–87]. The threat of high care costs and the risk of detention discourage women from attending health facilities, increasing the risk of preterm birth and related deaths [84]. Despite clearly violating human rights, this practice continues in many settings [88], highlighting the urgency and critical importance of implementing equity-enhancing UHC policies.

Partnership with women and parents of preterm babies requires specific require specific resources, such as beds for Kangaroo care [89], but facilities can partner with parents, whose experiences are valuable for helping to design these units. Parent–provider communication at the facility level is critical for preterm birth outcomes.

Strong leadership and management in health systems are critical to address the systemic barriers to respectful care [45]. Organizational issues – such as high workload,, poor supervision, low or inequitable pay, lack of autonomy, and workplace violence – disproportionally affect nurses and midwives [39, 90, 91]. Health workers are often blamed for shortcomings despite lacking adequate tools and support. Transformational leadership must prioritize investments in the health workforce, promote midwives and nurses into leadership roles, and improve accountability and supervision. Participatory learning approaches are also needed to develop context-specific solutions that advance respectful care, particularly for preterm birth, and to ensure providers are empowered and equipped to deliver high-quality care [92].

### Community level: empowerment and participation matter

There are multiple approaches to community-level engagement for rights and accountability. Social accountability programmes typically entail communities assessing the quality of care in health facilities against an agreed standard, and then engaging in dialogue and collective action with facility staff and leadership to negotiate improvements and accountability [93]. Most efforts to date have focused on accountability for clients of health services or their communities, with district or national level health authorities (usually the Ministry of Health) responsible for delivering on the right to high-quality maternal and newborn health care. For their part, States and those in charge of health systems can enable and institutionalize participation and by establishing community advisory committees or hospital community boards. Likewise, healthcare providers and communities can organize to demand improvements in their working conditions, and by extension, their ability to provide respectful, accountable care.

**Fig. 7:**
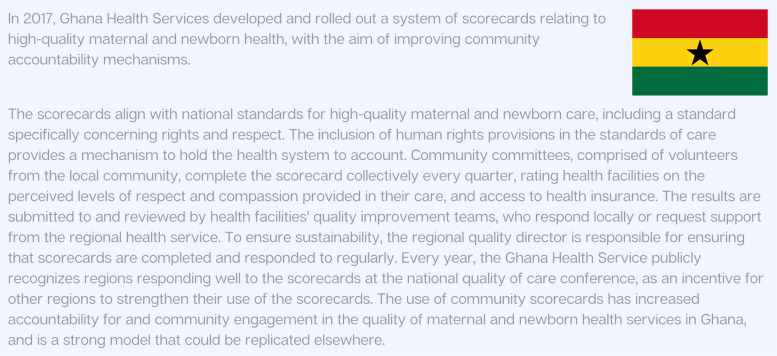
Country snapshot: Ghana’s quality of care community scorecards

Community scorecards are another initiative that can improve the quality of care in health facilities by ensuring the accountability of health providers or government, as demonstrated in Ghana (Fig. 7). Other initiatives, such as community meetings, may solicit specific improvements that health facilities can make to better serve families at high risk or those caring for a preterm baby. Social and behaviour change communication campaigns can raise awareness of the needs and rights of preterm babies and their families [94].

Women’s support groups and peer support models, such as parent groups, reflect evidence-based strategies for improving health outcomes, preparedness after discharge and parents’ mental health [95–98]. Further understanding of different approaches and preferences in different contexts may strengthen and sustain these programmes, such as those in Kenya [99] and Ghana [100]. Women and parents can partner in research projects and in health system redesign and contribute their unique expertise [101]. There is a need to better measure meaningful community leadership and participation in health system improvement and strengthening, especially with regard the needs of preterm babies and their families.

### National level: laws, participation and accountability matter

States have a responsibility to adopt and implement robust domestic legislation, firmly aligned with ratified international and regional human rights instruments, relating to preterm birth, and monitor compliance and any violations taking place. Such legislation, grounded in international human rights law, provides the essential legal foundation for policies and interventions to uphold the right to health, family leave, breastfeeding and healthcare workers’ rights [75, 102, 103]. Comprehensive, multi-component policies that tackle multiple underlying factors can also contribute to more respectful and equitable care [104]. States must also actively monitor, document, remedy and respond to rights violations, which are often underreported, and establish strong data systems to ensure accountability [22]. Critically, States must protect women and families from the financial burden of preterm birth from direct out of pocket expenditure through comprehensive Universal Health Coverage (UHC) plans [105].

The social, environmental and other broader determinants of maternal and newborn health, such as pollution and racism-related stress, also need to be addressed [25]. States have a duty to actively monitor and document rights violations on individual and population levels. Multisectoral collaboration and social mobilization are necessary where violations have become normalized due to drivers perceived to be unchangeable, such as stigma, discrimination, corruption and rigid professional hierarchies [106–108].

At the national level, social participation and community-driven efforts to interpret global guidance and to demand accountability for implementation are key. Those affected by preterm birth should participate actively in the formulation and adoption of relevant national and subnational policies and practices.

When parents and healthcare professionals collaborate, combining their skills and experience, they can make meaningful progress that addresses inequalities and fosters respectful care [101]. Accountability mechanisms that enable public conversations about preterm-related rights, such as citizen hearings or public dialogues, should be adapted to context and implemented in a timely manner [109].

**Fig. 8:**
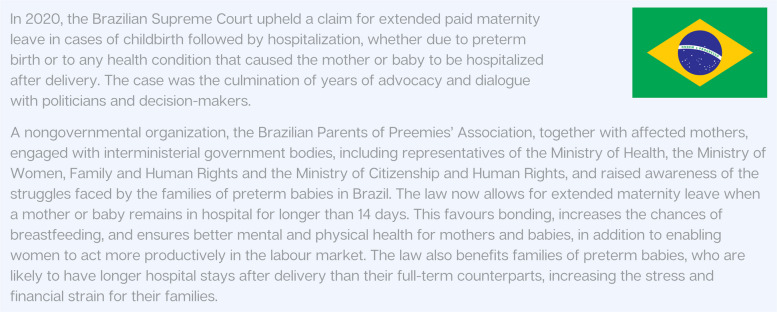
Country snapshot: maternity leave in Brazil

Advocacy days or months also provide opportunities for raising awareness and advancing norms around rights and respect relating to preterm birth, for example, World Prematurity Day, World Birth Defects Day and Pregnancy and Infant Loss Remembrance Month. Coordinated advocacy efforts can also lead to policy change, as demonstrated by the extension of the national maternity leave policy in Brazil (Fig. 8).

When national legal accountability mechanisms are exhausted, regional human rights bodies play an important role in monitoring human rights standards in maternity care and setting standards [110]. For example, the European Court of Human Rights (ECHR) has recognized the necessity of ensuring confidentiality and informed consent during facility-based childbirth in a case where medical students were observing a birthing woman without her consent [111]. The ECHR has found that separating a mother and a newborn at birth is an extremely harsh measure and that there need be unusually compelling reasons for such an action [112]. In 2023, the Inter-American Court of Human Rights explicitly held that “women have the right to live a life free of obstetric violence and States have the obligation to prevent it, punish it and refrain from practicing it, as well as to ensure that their agents act accordingly.” [113]

### Pivots

Rights-based approaches to preterm birth care have never been more acutely needed. Multilevel, multisectoral, and multistakeholder action is needed to address the systemic barriers preventing the realization of these rights by the most affected [25]. While all countries would greatly benefit from a wide range of interventions, four major shifts towards respectful and rights-based care should be prioritized. These shifts are recommended to create a more equitable, respectful, and rights-based framework and an enabling environment for quality preterm birth care, ensuring that systemic barriers are addressed and the rights of all individuals are upheld.

#### 1. Scale up respectful care for women, preterm babies and families

Realizing respectful care by scaling up RMC and FCC approaches will require whole systems to be reimagined and redesigned. Respectful care has not typically been front and centre in responses to global crises for women and preterm babies [114], and resources, capacity, and commitment to protect and fulfil rights are limited in many contexts. Additionally, increasing calls to localize and “decolonize” public health and humanitarian assistance are echoed by calls for greater community input into decisions, attention to rights, and consideration of the full spectrum of sexual, reproductive, maternal and newborn health in service delivery [115].

With “experience of care” embedded in global policies and guidelines, national and subnational policies and resources are needed to enable its implementation. For example, ensuring respectful care, including RMC and FCC, is integrated into future pandemic responses will be essential [16]. Likewise, financial protection, as part of UHC, needs to be in place for families affected by preterm birth.

#### 2. Empower and partner with women and families

Meaningful partnership and active participation are fundamental human rights principles. Women and affected families have a central role in the prevention and care of preterm babies. Protecting of their rights requires deliberate action and accountability, including mobilization through networks, digital technologies, social media, social accountability mechanisms and legal empowerment. Empowering and partnering with advocates, civil society organizations, and community-based initiatives, such as women’s groups and affected parents’ groups, are necessary to deliver preterm birth prevention and care that is both effective and supportive of rights. Policymakers and donors should exploring ways to better partner with, equip and finance grassroots organizations, creating platforms for them to voice their needs and concerns, and advocate for respectful care. Involving women and families of preterm babies in all aspects of care, as well as policy, budgetary, administrative, and judicial processes can ensure their needs are addressed, improves the quality of care and strengthens the implementation and sustainability of RMC and FCC.

#### 3. Protect healthcare providers’ rights

 The health service is responsible for delivering effectively, life-saving, harm-free care while upholding the rights of both those seeking and those providing care. To achieve this, all individuals working in health care must receive fair pay and work in safe, supportive and dignified environments. The additional demands of caring for preterm babies, supporting families facing fear and grief, and the risk of secondary trauma, should be acknowledged, and programmes established to strengthen health worker coping skills and resilience. Eliminating workplace harassment and the safeguarding the rights of health workers’ are urgently priorities, requiring clear accountability mechanisms through which the State can be held responsible. Both legal and social measures are essential, including explicit reporting mechanisms and transparent consequences for violations at community, facility and national levels. For example, healthcare providers must be able to raise issues, such as stock outs, without fear of retaliation. In addition, healthcare providers and facilities need to be protected from being targeted during conflicts.

#### 4. Strengthen inclusive, transparent and data-driven policy action and accountability

Strengthening accountability in the health system is essential to improving care for women and preterm babies, as well as the working conditions of healthcare providers. Transparent, well-defined accountability mechanisms must be established at all levels: among providers, within facilities and throughout the health system. To achieve this, stronger and more comprehensive data are needed to drive action to improve preterm birth outcomes, including qualitative and quantitative indices, benchmarks and tools to guide action and track progress. Standard indicators for RMC and FCC should be developed and agreed, and routinely collected, alongside maternal and newborn health indicators. These data must be embedded within national health information systems to inform strategies for improving outcomes. Clear mechanisms addressing human rights violations in maternity and neonatal care need to be established and communicated to all stakeholders. Governments must take decisive action in response to every violation to ensure accountability, build trust, and drive sustainable improvements in care.

## Conclusion

Advancing the preterm birth agenda requires a comprehensive human rights approach, one that builds on existing international, regional, and national laws, clinical guidelines, and the advocacy and action of communities working to promote sexual, reproductive, maternal and child health and rights for providers, women, babies and their families. Rethinking health and social systems to fully integrate human rights demands fundamental shifts in how policies and practices for preterm birth prevention and care are designed and implemented. This approach requires more than simply adding human rights principles as an afterthought; it calls for embedding them at the core of every policy, service, and interaction. Achieving this vision will need bold, coordinated multi-stakeholder action and strong partnerships between mothers, healthcare professionals, families, communities and systems that support them. A rights-based approach will drive holistic, sustainable improvements that benefit those everyone affected by preterm birth.

## Supplementary Information

Additional file 1

## Data Availability

All data is available in the paper or in supplementary files. Additional information is available at www. Borntoosoonaction.org
