# Supplementary material for: Born Too Soon: Progress and priorities for respectful and rights-based preterm birth care
Source: Reprod Health. 2025 Jun 23;22(Suppl 2):112. doi: 10.1186/s12978-025-02042-w (PMC12186350; doi:10.1186/s12978-025-02042-w)
Supplement: Supplementary file 1 — Additional file 1 [file 12978_2025_2042_MOESM1_ESM.docx]

Appendix 1: Mapping of human rights related to preterm birth

**Key Human Rights Conventions relevant preterm birth and related rights**

| *Global*   - Convention on the Rights of the Child (1989) - Convention on Elimination of all forms of Discrimination against Women (1979) - International Covenant on Civil and Political Rights (1966) - International Covenant on Economic, Social and Cultural Rights (1966) - Convention on the Rights of Persons with Disabilities (2006) - Convention on the Elimination of All Forms of Racial Discrimination (1965) - International Labor Organization Conventions   *Regional*   - The African Charter on Human and People’s Rights (1981) - The African Charter on the Rights and Welfare of the Child (1990) - The American Convention on Human Rights (1969) - The Inter-American Convention on the Prevention, Punishment, and Eradication of Violence against Women (1994) - The European Convention on Human Rights and Biomedicine (1997) |
| --- |

Illustrative relevant articles to preterm birth:

| Human Right | Legal Foundation | Text |
| --- | --- | --- |
| Social protection | ICESCR | Art. 10 “Special protection should be accorded to mothers during a reasonable period before and after childbirth. During such period working mothers should be accorded paid leave or leave with adequate social security benefits” |
| Social protection | CEDAW | Art. 11 “maternity leave with pay or with comparable social benefits without loss of former employment, seniority or social allowances” “provision of the necessary supporting social services to enable parents to combine family obligations with work responsibilities and participation in public life, in particular through promoting the establishment and development of a network of child-care facilities” |
| Social protection | CRC | Art. 26 “for every child the right to benefit from social security, including social insurance” |
| Health | ICESCR | Art. 12 “enjoyment of the highest attainable standard of physical and mental health”; “reduction of the stillbirth-rate and of infant mortality and for the healthy development of the child” |
| Health | CEDAW | Art 12 “ensure to women appropriate services in connection with pregnancy, confinement and the post-natal period, granting free services where necessary, as well as adequate nutrition during pregnancy and lactation.” |
| Health | CRC | Art. 24 “enjoyment of the highest attainable standard of health and to facilities for the treatment of illness and rehabilitation of health”; “diminish infant and child mortality;” “ensure the provision of necessary medical assistance and health care;” “ensure appropriate pre-natal and post-natal health care for mothers.” |
| Health | CRPD | Art. 25 “persons with disabilities have the right to the enjoyment of the highest attainable standard of health without discrimination on the basis of disability.” “Provide those health services needed by persons with disabilities specifically because of their disabilities, including early identification and intervention as appropriate, and services designed to minimize and prevent further disabilities, including among children” |
| Family Planning | CEDAW | Art. 10 “Access to specific educational information to help to ensure the health and well-being of families, including information and advice on family planning.” |
| Equality and Nondiscrimination | ICCPR | Art. 26 “equal and effective protection against discrimination on any ground such as race, colour, sex, language, religion, political or other opinion, national or social origin, property, birth or other status” |
| Equality and Nondiscrimination | ICESCR | Art. 2 “without discrimination of any kind as to race, colour, sex, language, religion, political or other opinion, national or social origin, property, birth or other status.” |
| Equality and Nondiscrimination | CRC | Art. 2 “without discrimination of any kind, irrespective of the child's or his or her parent's or legal guardian's race, colour, sex, language, religion, political or other opinion, national, ethnic or social origin, property, disability, birth or other status.” |
| Equality and Nondiscrimination | CRC | Art. 23 “right of the disabled child to special care” |
| Equality and Nondiscrimination | CEDAW | Art. 12 “eliminate discrimination against women in the field of health care” |
| Equality and Nondiscrimination | CRPD | Art. 5 “prohibit all discrimination on the basis of disability and guarantee to persons with disabilities equal and effective legal protection against discrimination on all grounds”  Art. 25 “ensure access for persons with disabilities to health services that are gender-sensitive, including health-related rehabilitation.” |
| Information | ICCPR | Art. 19 “ freedom to seek, receive and impart information” |
| Information | CRC | Art. 13 “ freedom to seek, receive and impart information” |
| Information | CRC | Art. 5 “respect the responsibilities, rights and duties of parents,… members of the extended family or community, … legal guardians or other persons legally responsible for the child, to provide … appropriate direction and guidance in the exercise by the child of the rights ..” |
| Informed consent | European Convention on Human Rights in Biomedicine | Art. 5 “An intervention in the health field may only be carried out after the person concerned has given free and informed consent to it. This person shall beforehand be given appropriate information as to the purpose and nature of the intervention as well as on its consequences and risks. The person concerned may freely withdraw consent at any time.”  Art. 6 “Where, according to law, a minor does not have the capacity to consent to an intervention, the intervention may only be carried out with the authorisation of his or her representative or an authority or a person or body provided for by law.” |
| Respect and dignity | ICCPR | Art. 17 “unlawful attacks on his honour and reputation” |
| Respect and dignity | CRC | Art. 16 “unlawful attacks on his or her honour and reputation” |
| Respect and dignity | CRC | Art. 23 “mentally or physically disabled child should enjoy a full and decent life, in conditions which ensure dignity, promote self-reliance and facilitate the child's active participation in the community.” |
| Respect and dignity | African Charter on the Rights and Welfare of the Child | Art. 13 “Every child who is mentally or physically disabled shall have the right to special measures of protection in keeping with his physical and moral needs and under conditions which ensure his dignity, promote his self-reliance and active participation in the community.” |
| Privacy | ICCPR | Art. 17 “arbitrary or unlawful interference with his privacy, family” |
| Privacy | CRC | Art. 12 “arbitrary or unlawful interference with his or her privacy, family” |
| Privacy | CRDP | Art. 22 “protect the privacy of personal, health and rehabilitation information of persons with disabilities on an equal basis with others” |
| Privacy | European Convention on Human Rights in Biomedicine | Art. 10 “Everyone has the right to respect for private life in relation to information about his or her health. Everyone is entitled to know any information collected about his or her health. However, the wishes of individuals not to be so informed shall be observed.” |
| No separation | CRC | Art. 9 “a child shall not be separated from his or her parents against their will” |
| Nutrition | CEDAW | Art. 12 “States Parties shall ensure to women …  adequate nutrition during pregnancy and lactation." |
| Nutrition | CRC | Art. 24 "ensure that all segments of society, in particular parents and children, are informed, have access to education and are supported in the use of basic knowledge of child health and nutrition, the advantages of breastfeeding, hygiene and environmental sanitation and the prevention of accidents” |
| Nutrition | CRPD | Art 25. “Prevent discriminatory denial of health care or health services or food and fluids on the basis of disability.” |
| Best interest of the child | CRC | Art. 3 “In all actions concerning children, whether undertaken by public or private social welfare institutions, courts of law, administrative authorities or legislative bodies, the best interests of the child shall be a primary consideration.” |
| Decent work and Fair Pay | ICESCR | Art. 7 “Fair wages and equal remuneration for work of equal value without distinction of any kind, in particular women being guaranteed conditions of work not inferior to those enjoyed by men, with equal pay for equal work.” “Safe and healthy working conditions.” |
| Freedom of association | ICCPR | Art. 22 “the right to freedom of association with others, including the right to form and join trade unions for the protection of his interests.” |
| Freedom of association | ILO Convention No. 87 | Freedom of Association and Protection of the Right to Organize. |
| Collective Bargaining | ILO Convention No. 98 | Right to Organize and Collective Bargaining. |
| Equal remuneration | ILO Convention No. 100 | Equal Remuneration Convention |
| Discrimination in employment | ILO Convention No. 111 | Discrimination (Employment and Occupation) Convention |
| Occupational Safety | ILO Convention No. 155 | Occupational Safety and Health Convention |
| Freedom from Harassment | ILO Convention No. 190 | Art. 4 “right of everyone to a world of work free from violence and harassment.” |

Mapping of International and Regional Human Rights and Instruments Pertaining to the Rights of Women, Newborns and Healthcare Workers to demonstrate overlapping and intersecting rights

| **Human Right** | **International Human Rights Instruments** | | | | | |
| --- | --- | --- | --- | --- | --- | --- |
| Right to Health | ICESCR | CEDAW | CRC | CRPD |  |  |
| Right to Equality and Nondiscrimination | ICCPR | ICESCR | CEDAW | CRC | CRPD | ICERD |
| Right to Social Protection | ICESCR | CEDAW | CRC | CRPD | ICERD |  |
| Right to Information | ICCPR | CEDAW | CRC | CRPD |  |  |
| Right to dignity and privacy | ICCPR | CRC | CRPD |  |  |  |
| Right to food and nutrition | ICESCR | CEDAW | CRC | CRPD |  |  |
| Right to decent work and fair pay | ICESCR | ICERD | CRPD | CEDAW | ILO Convention No. 100 | ILO Convention No. 190 |
| Right to freedom of association and forming a union | ICCPR | ICERD | CRPD | ILO Convention No. 87 | ILO Convention No. 98 |  |

Key:

CRC: Convention on the Rights of the Child (1989)

CEDAW: Convention on Elimination of all forms of Discrimination against Women (1979)

ICCRP: International Covenant on Civil and Political Rights (1966)

ICESCR: International Covenant on Economic, Social and Cultural Rights (1966)

CRPD: Convention on the Rights of Persons with Disabilities (2006)

ICERD: Convention on the Elimination of All Forms of Racial Discrimination (1965)

ILO: International Labor Organization Conventions

(1-7)

1. United Nations. Convention on the Rights of the Child, Resolution 44/25,. United Nations General Assembly (1989). Available from: <https://www.ohchr.org/en/instruments-mechanisms/instruments/convention-rights-child>

2. United Nations. Convention on the Elimination of All Forms of Discrimination against Women, General Assembly resolution 34/180 (1979). Available from: <https://www.ohchr.org/sites/default/files/Documents/ProfessionalInterest/cedaw.pdf>

3. United Nations. International Covenant on Civil and Political Rights, General Assembly resolution 2200A (XXI). (1966). Available from: <https://www.ohchr.org/sites/default/files/ccpr.pdf>

4. United Nations. International Covenant on Economic, Social and Cultural Rights, General Assembly resolution 2200A (XXI). (1966). Available from: <https://www.ohchr.org/en/instruments-mechanisms/instruments/international-covenant-economic-social-and-cultural-rights>

5. United Nations. Convention on the Rights of Persons with Disabilities, A/RES/61/106. United Nations General Assembly (2006). Available from: <https://www.ohchr.org/en/instruments-mechanisms/instruments/convention-rights-persons-disabilities>

6. United Nations. International Convention on the Elimination of All Forms of Racial Discrimination, UN General Assembly resolution 2106. (1965). Available from: <https://www.ohchr.org/sites/default/files/cerd.pdf>

7. International Labour Organization [Internet]. Conventions and Recommendations 2023 [cited 26 March 2023.]. Available from: <https://www.ilo.org/global/standards/introduction-to-international-labour-standards/conventions-and-recommendations/lang--en/index.htm>.
